# A Rare Case of Adult Poorly Differentiated Chordoma of the Skull Base With Rapid Progression and Systemic Metastasis: A Review of the Literature

**DOI:** 10.7759/cureus.51605

**Published:** 2024-01-03

**Authors:** Keisuke Harada, Naoki Shinojima, Haruaki Yamamoto, Mai Itoyama, Daichi Uchida, Yuji Dekita, Satoru Miyamaru, Hiroyuki Uetani, Yorihisa Orita, Yoshiki Mikami, Kisato Nosaka, Toshinori Hirai, Akitake Mukasa

**Affiliations:** 1 Department of Neurosurgery, Kumamoto University Hospital, Kumamoto, JPN; 2 Department of Neurosurgery, Saiseikai Kumamoto Hospital, Kumamoto, JPN; 3 Department of Otolaryngology-Head and Neck Surgery, Kumamoto University Hospital, Kumamoto, JPN; 4 Department of Radiosurgery, Kumamoto Radiosurgery Clinic, Kumamoto, JPN; 5 Department of Diagnostic Radiology, Faculty of Life Sciences, Kumamoto University, Kumamoto, JPN; 6 Department of Diagnostic Pathology, Kumamoto University Hospital, Kumamoto, JPN; 7 Department of Cancer Treatment Center, Kumamoto University Hospital, Kumamoto, JPN; 8 Department of Hematology, Rheumatology, and Infectious Diseases, Kumamoto University Hospital, Kumamoto, JPN

**Keywords:** skull base, ki-67 labeling index, systemic metastasis, rapid progression, adult, poorly differentiated chordoma

## Abstract

Chordoma is a rare tumor that arises from chordal tissue during fetal life. Recently, the concept of poorly differentiated chordoma, a subtype of chordoma characterized by loss of *SMARCB1*/*INI1* with a poorer prognosis than conventional chordomas, was established. It predominantly occurs in children and is rare in adults. Here, we report a rare adult case of poorly differentiated chordoma of the skull base with a unique course that rapidly systemically metastasized and had the shortest survival time of any adult chordoma reported to date.

The patient was a 32-year-old male with a chief complaint of diplopia. MRI showed a widespread neoplastic lesion with the clivus as the main locus. Endoscopic extended transsphenoidal tumor resection was performed. Pathological findings showed that the tumor was malignant, and immunohistochemistry revealed a Ki-67 labeling index of 80%, diffusely positive brachyury, and loss of *INI1 *expression. The final diagnosis was poorly differentiated chordoma. Postoperatively, the residual tumor in the right cavernous sinus showed rapid growth. The patient was promptly treated with gamma knife three fractions. The residual tumor regressed, but the tumor developed systemic metastasis in a short period, and the patient died seven months after diagnosis.

This report of a rapidly progressing and fatal adult poorly differentiated chordoma shows the highest Ki-67 labeling index reported to date. Prompt multidisciplinary treatment should be considered when the Ki-67 labeling index is high.

## Introduction

Chordomas are rare tumors that arise from fetal chordal tissue, i.e., notochordal tissue, and predominantly occur in adults [1,2]. They occur in the spine, particularly in the sacrum and skull base, and are difficult to remove entirely due to their infiltrative nature [3]. Median overall survival is reported to be less than 10 years [4]. Furthermore, according to the World Health Organization Classification of Tumors, Soft Tissue and Bone Tumors (fifth edition), poorly differentiated chordoma is reported as a subtype of high-grade chordoma. Its features are similar to those of conventional chordoma in that brachyury and epithelial antigen expression are maintained. However, unlike conventional chordoma, *SWI/SNF-related matrix-associated actin-dependent regulator of chromatin subfamily B member 1* (*SMARCB1*)/*integrase interactor 1 *(*INI*) expression is absent [5-7], the disease predominantly affects children, and the prognosis is poor with an average overall survival of approximately 50 months [8,9].

We experienced an adult case of poorly differentiated chordoma of the skull base with rapid local progression and systemic metastasis, resulting in uncontrolled disease and the shortest survival of any adult case reported to date. Here, a poorly differentiated chordoma with systemic metastasis is discussed with a review of the literature.

## Case presentation

Clinical summary

**Figure 1:**
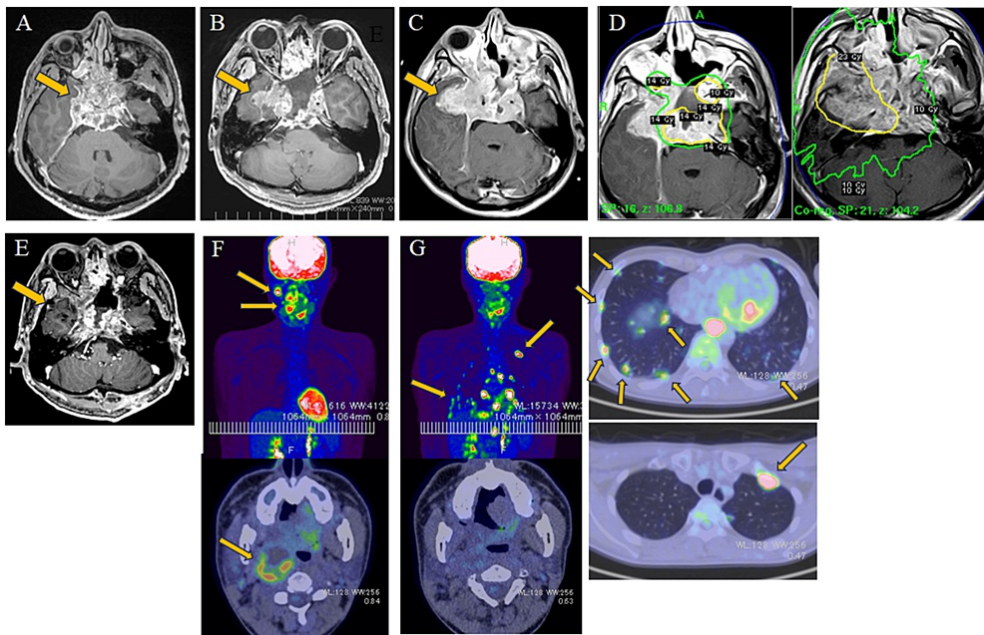
Images during the course of the disease. MRI showing preoperative findings (A), third day after surgery (B), and two weeks after surgery (C). A 14-Gy dose was delivered to the tumor margin at the 40% isodose (yellow line) for a lesion from the cavernous sinus to the left middle cranial fossa (D left panel) and a 23-Gy dose was delivered to the tumor margin at the 40% isodose (yellow line) for a lesion from the right side of the clivus to the right middle cranial fossa (D right panel). Two months after irradiation by a gamma knife (E). Fludeoxyglucose-18 positron emission tomography three months after surgery (F) and 5.5 months after surgery (G).

A 32-year-old male presented to his family physician with a chief complaint of diplopia. He presented with right III, IV, V1, VI, and VII cranial nerve palsy. MRI showed neoplastic lesions involving the clivus, sphenoid sinus, anterior ethmoid sinus, sella region, bilateral cavernous sinuses, and right internal and external pterygoid muscles (Figure 1A). The patient was referred to our hospital on suspicion of sinonasal carcinoma based on the rapid growth and extension of the tumor. Endocrinologic examination revealed secondary adrenal insufficiency due to anterior pituitary hypofunction. His symptoms improved after starting hydrocortisone replacement at 30 mg/day. No diabetes insipidus was observed. Endoscopic extended transsphenoidal tumor resection was performed. Intraoperative findings showed that the tumor was very hemorrhagic. The extent of the resection was a partial resection. Postoperatively, the residual tumor in the right cavernous sinus increased rapidly and extended into the middle cranial fossa, compressing the right temporal lobe (Figures 1B, 1C). Eventually, his right eye went blind due to occlusion of the superior ophthalmic vein. The final histopathological diagnosis was poorly differentiated chordoma. The patient was promptly treated with a gamma knife in the hope of local control by high-dose irradiation. Because the target volume was too large for the gamma knife to irradiate in a single fraction, multifractionated irradiation was performed. A lesion (planning target volume (PTV) of 236.7 mL) from the cavernous sinus to the left middle cranial fossa was irradiated with a marginal dose of 14.0 Gy (central dose of 35 Gy) in two fractions (equivalent to a marginal dose of 21.4 Gy in terms of a single fraction) (Figure 1D left). A single fraction of 23.0 Gy marginal dose (57.5 Gy central dose) was given to a lesion (PTV of 123.9 mL) in the right middle cranial fossa from the right side of the clivus (Figure 1D right). Because of the prolonged irradiation time, the patient developed severe fatigue due to relative adrenal insufficiency, but an additional 100 mg of hydrocortisone was administered on each irradiation day. The patient managed to complete a total of three days of irradiation. The high-dose irradiation resulted in marked shrinkage of the residual tumor and improvement of symptoms (Figure 1E). However, a whole-body examination with fludeoxyglucose-18 positron emission tomography (FDG-PET) revealed abnormal accumulation in cervical lymph nodes and Lubyer’s lymph nodes outside the gamma knife irradiation field (Figure 1F). The patient underwent additional stereotactic radiotherapy to these metastatic lymph nodes. Subsequent FDG-PET showed that the abnormal accumulation in the additionally irradiated cervical lymph nodes had disappeared, but numerous abnormal accumulation images were observed in the lungs and mediastinal lymph nodes (Figure 1G), indicating systemic metastasis. Microsatellite instability (MSI) was examined, but immunotherapy could not be given because MSI was low. Although chemotherapy was planned, the disease progressed more rapidly than expected and the patient died seven months after diagnosis.

Pathological findings

**Figure 2:**
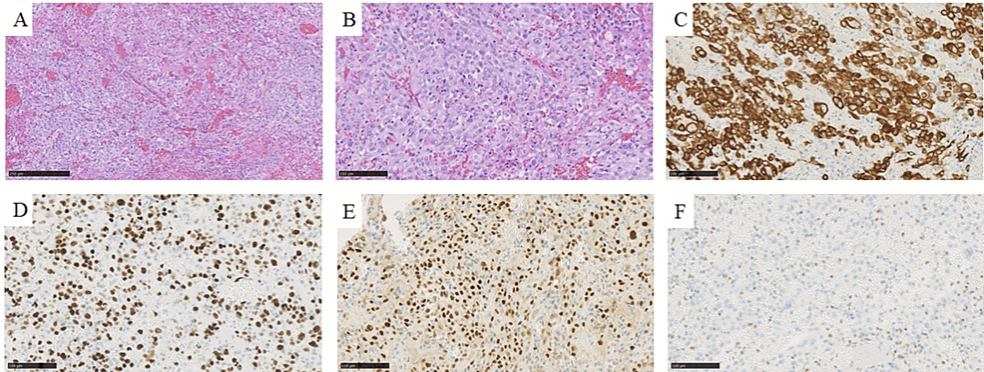
Histopathology findings. Hematoxylin and eosin staining at 100× (A), 200× (B), and immunostaining (all 200×) for cytokeratin (AE1/AE3) (C), Ki-67 (D), brachyury (E), and *INI1* (*SMARCB1*) (F).

Pathology revealed epithelial-like atypical cells with a pale eosinophilic to eosinophilic cytoplasm proliferated in sheets or foci. Nuclear atypia was strong, and the majority of the cells had a component without an intervening mucus matrix. The highest mitotic count was 2-3 cells/10 high-power field (field of view 0.55 mm) with necrosis. A small number of components with mild cellular atypia with mucus matrix inclusions were also observed (Figures 2A, 2B). Immunohistochemistry was performed on formalin-fixed paraffin-embedded specimens with validation of positive and negative controls according to our previously reported protocol [10,11]. Immunostaining revealed that these atypical cells were diffusely positive for cytokeratin (AE1/AE3), and the Ki-67 labeling index was approximately 80%, suggesting malignancy (Figures 2C, 2D). Additionally, the tumor cells manifested diffuse positivity for brachyury (Figure 2E), a marker for chordoma, and loss of expression of *INI1* (*SMARCB1*) (Figure 2F). According to the World Health Organization Classification of Tumors, Soft Tissue and Bone Tumors (fifth edition), the final diagnosis of poorly differentiated chordoma was made.

## Discussion

Survival analysis of poorly differentiated chordoma based on previous reports

We hypothesized that the prognosis of poorly differentiated chordoma with metastases, as in our case, would be poor and performed a prognostic analysis.

Methods

We searched PubMed for reports of poorly differentiated chordomas reported up to 2022 using the keyword “poorly differentiated chordoma.” A total of 87 cases with *INI1 *loss and positive brachyury were extracted [1,6,12-30]. Kaplan-Meier survival statistics and log-rank test were performed on 34 cases, including the present case, to examine overall survival with and without metastases. All statistical analyses were performed using BellCurve for Excel (Social Survey Research Information Co., Ltd. Tokyo, Japan).

Results

**Figure 3:**
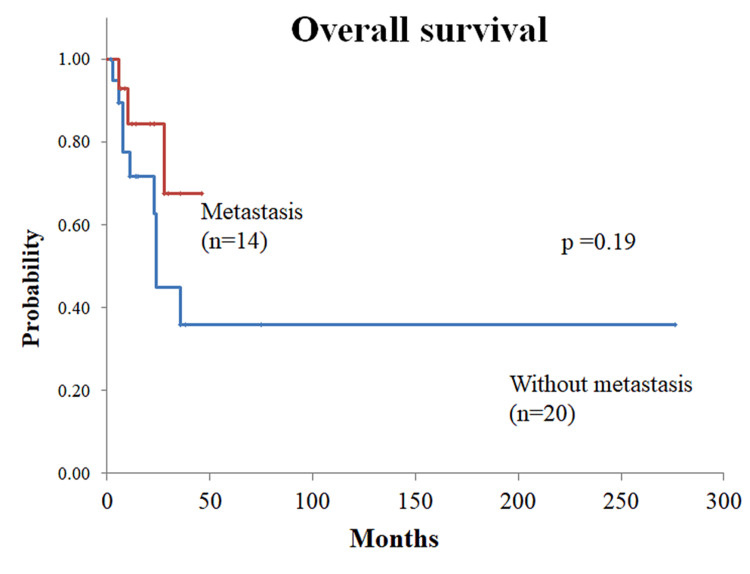
Comparison of overall survival between poorly differentiated chordoma with metastasis (red line, n = 14) and without (blue line, n = 20). The Kaplan-Meier survival curve showed that the median overall survival was not reached for patients with metastases and the median overall survival for non-metastatic cases was 24 months. A log-rank test has no significant differences between them (p = 0.19).

As shown in Table 1, gender was known in 86 of the 88 cases, with 41 males and 45 females. Age was known in 79 cases and ranged from 0 to 52 years, and 54 cases (68.4%) were younger than 15 years of age. The outcome was known in 56 cases; 33 cases were alive, and 23 were deceased due to the primary disease. Overall survival ranged from 0.3 to 276 months. Of the 23 patients who died of the original disease, only two (8.7%) were aged 15 years or older. The present case with distant metastases and a 22-year-old woman without metastases. The adult case with the shortest survival time was the present case. Information on metastasis was obtained from 53 patients, 29 (54.7%) had metastasis, and 24 (45.3%) did not. Overall survival was available for 34 of the 53 patients, 14 with metastases (three of whom died) and 20 without metastases (nine of whom died). Kaplan-Meier survival curve analysis showed no significant difference in survival by metastatic status (p = 0.19, log-rank test) (Figure 3). However, the present case had the shortest survival time among the deceased patients with metastases. The Ki-67 labeling index was reported in 18 patients, ranging from 2% to 80%, with the present case having the highest value reported.

**Table 1:** Reported cases of poorly differentiated chordoma in the literature. OS: overall survival; AWD: alive with disease; DWD: deceased with disease; NED: no evidence of disease; n.a.: data not available; DR: distant recurrence

Author, year	Sex	Age (years)	Location	Outcome	OS (months)	Metastasis	Metastasis location	Ki-67 (%)
Mobley et al., 2010 [6]	Female	11	Sacrum	DWD	28	Yes	Lung, liver	n.a.
	Female	22 months	Clivus	AWD	12	Yes	Spine	n.a.
	Male	7	Clivus	DWD	8	No	-	n.a.
	Female	3	Clivus	n.a.	n.a.	No	-	n.a.
Chavez et al. 2014, [12]	female	2	Craniocervical junction	n.a.	n.a.	n.a.	n.a.	n.a.
Renard et al., 2014 [13]	Female	27 months	Clivus	AWD	9	Yes	pleura	n.a.
	Male	2	Clivus	DWD	n.a.	No	-	n.a.
Yadav et al., 2014 [14]	Male	7	Clivus	AWD	2	No	-	5
	Female	8	Clivus	AWD	75	No	-	2
	Male	4	Clivus	n.a.	n.a.	No	-	6
	Male	10	Clivus	DWD	3	No	-	3
	Male	6	Clivus	DWD	n.a.	No	-	2
	Male	18	Clivus	AWD	6	No	-	12~13
	Female	2	Cervical	DWD	n.a.	No	-	3
Hasselblatt et al., 2016 [15]	4 boys and 3 girls	Median 7 (range 1–12)	Clivus	DWD(6/7), AWD (1/7)	9 (median)	n.a.	n.a.	n.a.
			Clivus			n.a.	n.a.	n.a.
			Clivus			n.a.	n.a.	n.a.
			Clivus			n.a.	n.a.	n.a.
			Clivus			n.a.	n.a.	n.a.
			Clivus			n.a.	n.a.	n.a.
			Clivus			n.a.	n.a.	n.a.
Antonelli et al., 2017 [16]	Male	17	Clivus	AWD	2 years	n.a.	n.a.	n.a.
	Male	11	Clivus	n.a.	n.a.	n.a.	n.a.	n.a.
	Male	8	Clivus	DWD	1 year	n.a.	n.a.	n.a.
	Female	16	Cervical	NED	10	n.a.	n.a.	n.a.
Cha et al., 2018 [17]	Female	6	Clivus	AWD	36	No	-	15
	Male	7	Clivus	AWD	15	No	-	7–8
Rekhi et al., 2018 [18]	Female	42	Cervical	AWD	n.a.	Yes	Lung	40
	Male	4	Cervical	AWD	36	No	-	60
Owosho et al., 2018 [19]	Male	3	Clivus, Cervical	n.a.	n.a.	n.a.	n.a.	n.a.
	Female	4	Clivus	n.a.	n.a.	n.a.	n.a.	n.a.
	Female	7	Dura mater	n.a.	n.a.	n.a.	n.a.	n.a.
	Male	17	Cervical	NED	14	No	-	n.a.
	Female	2	Clivus	AWD	28	Yes	Lung	n.a.
	Female	25	Sacrum	AWD	23	Yes	Lung, pericardium	n.a.
	Female	8	Cervical	AWD	23	Yes, DR	Rib, humerus, lung	n.a.
	Female	21	Cervical	AWD	14	Yes, DR	rib	n.a.
	Female	3	Clivus	n.a.	n.a.	Yes, DR	n.a.	n.a.
Shih et al., 2018 [20]	Male	16	Coccyx	n.a.	n.a.	n.a.	n.a.	10–15 in 2 of 17
	Male	19	Cervical	n.a.	n.a.	n.a.	n.a.	
	Female	18	Cervical (C2–3)	AWD	46	Yes	n.a.	
	Female	3	Skull base	DWD	10	Yes	n.a.	
	Male	20	Cervical (C5)	NED	36	Yes	n.a.	
	Male	17	Cervical (C2–3)	Alive; unknown disease status	6	No	-	
	Female	22	Cervical (C3–5)	DWD	23	No	-	
	Female	20	Sacrum	AWD	0.3	n.a.	n.a.	
	Male	2	Clivus	n.a.	n.a.	n.a.	n.a.	
	Female	1	Skull base clivus	AWD	30	Yes	n.a.	
	Female	1	Clivus	AWD	11	No	-	
	Female	5	Cervical (C1–2)	NED	12	n.a.	n.a.	
	Female	9	Clivus	NED	9	n.a.	n.a.	
	Female	17	Clivus	AWD	14	No	-	
	Female	29	Cervical (C6–7)	NED	29	n.a.	n.a.	
	Female	7	Clivus, Cervical (C1–2)	NED	11	n.a.	n.a.	
	Male	11	Skull base, clivus	Alive; unknown disease status	38	No	-	
Buccoliero et al., 2019 [21]	Male	23 months	Clivus	n.a.	n.a.	No	-	30
	Male	30 months	Clivus	DWD	3 years	No	-	30
	Female	12	Cervical (C2–5)	DWD	2 years	No	-	10
	Female	3 months	Cerebellar left	DWD	2 years	No	-	30
Gounder et al., 2019 [22]	Female	25	Sacrum	n.a.	n.a.	Yes	Lung	n.a.
Jaber et al., 2019 [1]	Male	30	Cervical (C6)	n.a.	n.a.	Yes	Lung	n.a.
Curcio et al., 2021 [23]	Male	43	Sacrum	n.a.	n.a.	Yes	Lung	n.a.
Kohashi et al., 2021 [24]	Male	7	Clivus	DWD	17	n.a.	n.a.	n.a.
	Male	6 months	Clivus	DWD	20	n.a.	n.a.	n.a.
	Male	3	Cervical	n.a.	n.a.	n.a.	n.a.	n.a.
	Female	5	Clivus	n.a.	n.a.	n.a.	n.a.	n.a.
	Female	2	Clivus	n.a.	n.a.	n.a.	n.a.	n.a.
	male	9	Clivus	n.a.	n.a.	n.a.	n.a.	n.a.
Rekhi et al., 2021 [25]	Female	1.5	Clivus extending to sella	n.a.	8	No	-	n.a.
	Male	2	Coccyx	NED	23yaers	No	-	n.a.
	Female	29	Paravertebral region (L3–5)	n.a.	n.a.	Yes	Lung, rib, femoral head acetabulum, brain	n.a.
	Female	14	Thoracic vertebra (T2–4)	n.a.	n.a.	Yes	Lung	n.a.
	Male	15	Paravertebral space. Cervical (C2–3)	n.a.	n.a.	No	-	n.a.
	Male	1	Sphenoethmoidal mass	n.a.	n.a.	No	-	n.a.
	Male	3	Cervical (C1–2)	n.a.	n.a.	No	-	n.a.
	Female	6	Skull base	n.a.	11	No	-	n.a.
	Male	1.5	Clivus	n.a.	6	No	-	n.a.
Schaefer et al., 2021 [26]	Female	21	NA	n.a.	n.a.	Yes	Pleura fluid	n.a.
Wen et al., 2021 [27]	Female	21	Left elbow	AWD	7	Yes	Pleura, mediastinal lymph nodes	n.a.
	Male	52	Left knee	AWD	21	Yes	Inguinal lymph node, rib, pleura	n.a.
Williamson et al., 2021 [28]	NA	Pediatric	Cervical (C1)	n.a.	n.a.	Yes	Dens, clivus, left occipital condyle	n.a.
	NA	pediatric	Cervical (C1–2)	n.a.	n.a.	Yes	Lung	n.a.
Zhao et al., 2021 [29]	Female	2	Clivus, Cervical (C2)	DWD	6	n.a.	n.a.	n.a.
	Male	2	Clivus	DWD	6	n.a.	n.a.	n.a.
	Female	6	Clivus	AWD	16	n.a.	n.a.	n.a.
	Male	13	Sacrum		n.a.	n.a.	n.a.	n.a.
Yasue et al., 2022 [30]	Female	2	Clivus	n.a.	n.a.	Yes	Left upper arm, right iliac bone	n.a.
Harada et al., 2023 Present case	Male	32	Clivus	DWD	7	Yes	Lung, lymph node	80

Discussion

Reaching a diagnosis of poorly differentiated chordoma is not always easy because the concept of this disease is not yet widely disseminated [1]. The pathology of the present case revealed a highly atypical component with little myxoid stroma and a dense proliferation of epithelial-like atypical cells. Rhabdoid-like atypical cells with a deviated nucleus and eosinophilic cytoplasm were also partially observed. Immunostaining showed that the patient was keratin-positive and S100 protein-negative, a feature similar to rhabdoid meningiomas and atypical teratoid/rhabdoid tumors (AT/RT). Furthermore, in this case, *SMARCB1*/*INI1 *was lost, and mutations or deletions of *SMARCB1 *have been reported in malignant rhabdoid tumors, which are also absent in AT/RT [6,13,17,20,21,31,32]. An important distinction between these *SMARCB1*/*INI1*-expressing tumors and this case is the positive brachyury, a specific finding that indicates chordoid differentiation and is positive in all subtypes of chordomas. In the present case, the positive brachyury was sufficient to conclude that the patient had chordoma. Conventional chordoma does not show loss of *SMARCB1*/*INI1* expression, and chordomas that show loss of *SMARCB1*/*INI1 *expression are considered poorly differentiated chordoma [2,6,16,19]. Thus, the diagnosis of poorly differentiated chordoma was made in this case.

A total of 87 cases of poorly differentiated chordoma have been reported in the literature through 2022, and Table 1 includes a total of 88 cases, including our case. Poorly differentiated chordoma is most commonly diagnosed in early childhood, the average age of onset is about 10 years [8]. Similarly, our literature review showed that about 70% of cases were under the age of 15, indicating that poorly differentiated chordoma is more common in children. Thus, the present case was diagnosed in the 30s, which is extremely rare. Metastasis of poorly differentiated chordoma has rarely been reported, accounting for about 20% of cases [20]. Of the cases with known metastases, only about half (54.7%) had metastases, but the proportion with metastases is potentially higher because some cases have not been systemically examined and clinical data are lacking. In this case, stereotactic radiosurgery was performed as treatment, and the tumor within the irradiated field was reduced. However, systemic metastases developed, and the patient died approximately seven months after tissue diagnosis, the shortest overall survival reported to date.

We hypothesized that poorly differentiated chordoma with metastasis, as in this case, would have a poor prognosis and performed a prognostic analysis. However, we did not find a statistically significant difference in survival rate between patients with and without metastases. This may be because only three of the 14 patients with metastases were confirmed dead, and the others were censored cases, so the data were insufficient. The overall survival of the other two patients was 10 and 28 months, respectively, and the overall survival of this patient was the shortest at seven months. Regarding the proliferative potential of poorly differentiated chordoma, the Ki-67 labeling index is generally reported to be 10-15% [8,14], but in this case, the Ki-67 labeling index was extremely high at approximately 80%, the highest value reported. Therefore, the reason for the shortest survival in our case may be related to the highest Ki-67 labeling index reported to date, and prompt multidisciplinary treatment should be considered when the Ki-67 labeling index is very high. Carbon ion beam therapy has received much attention as radiation therapy for chordoma, but there are few reports of proton or heavy ion beams for poorly differentiated chordoma [33-35], and it is not known how effective these treatments are. Chemotherapy is not an effective treatment either [8,9,28]. Immunotherapy, including an immune checkpoint inhibitor, was not administered due to low MSI, and a cancer gene panel test was attempted for TMB, but the patient was too advanced in stage to submit the test. Cancer gene panel testing should have been done at an earlier stage. As we previously reported [11], the development of immunotherapy and molecular-targeted agents and the collection of therapeutic data will be important in the future [9].

## Conclusions

This report of a rapidly progressing and fatal adult poorly differentiated chordoma showed the highest Ki-67 labeling index reported to date. Prompt multimodality treatment, including immunotherapy, should be considered in cases with a high Ki-67 labeling index because of the possibility of early systemic metastasis.
